# Management of a Periodontal Pocket Using a Removable Orthodontic Appliance and Nonsurgical Periodontal Therapy

**DOI:** 10.1155/2015/374850

**Published:** 2015-09-03

**Authors:** Serhat Köseoğlu, Ahmet Fidancıoğlu, Mehmet Sağlam, Levent Savran

**Affiliations:** ^1^Department of Periodontology, Faculty of Dentistry, İzmir Katip Çelebi University, Aydinlikevler Mahallesi, Cemil Meriç Bulvarı, 6780 Sokak No. 48, 35640 Çiğli/İzmir, Turkey; ^2^Private Practice, Konya, Turkey

## Abstract

*Purpose*. As documented in the literature, bony defects can be managed by an orthodontic approach. *Methods*. This case report describes the treatment of a bony defect caused by orthodontic malposition through phase I periodontal therapy and a simple removable orthodontic appliance used for the first time in a 20-year-old girl. *Results*. The periodontal pocket was reduced from 8 mm to 3 mm shortly after treatment. *Conclusion*. This case report concludes that orthodontic therapy can be used successfully in treatment of bony defects caused by mesially tilted molars.

## 1. Introduction

Orthodontic malposition may act as a local predisposing factor in periodontitis and induce periodontal pockets and infrabony defects because of uncontrolled plaque accumulation [1]. Several treatment procedures combined with scaling and root planing (SRP) have been presented to eliminate infrabony defects [2, 3], including the use of various bone graft or bone substitute materials, root surface conditioning, and growth factors for guided tissue regeneration (GTR) treatments [4].

Impaction of permanent second molars is a rare condition, but it usually causes periodontal problems when present. These clinical situations may occur because of alveolar arc length, tooth size, or axial inclination [5]. Unfavorable mesial inclination of a secondary molar not only causes functional disadvantages (e.g., extra-axial occlusal forces and lack of pontic space) but also leads to periodontal problems (e.g., compressed marginal gingiva, plaque retention, and acute-angled osseous contour). This clinical incident predisposes periodontal pockets and infrabony defects between the proximal sites of the first and secondary molars [6].

The local factors that cause periodontal breakdown should be eliminated before periodontal therapy to achieve successful treatment. Previous studies show that, in orthodontic malposition cases, surgical periodontal treatment alone is inadequate [7]. To eliminate the underlying etiology, orthodontic uprighting has been suggested to maintain arch integrity [8] and to improve the periodontal status with increased ease of plaque removal and reduced occlusal trauma [9]. Moreover, a better gingival contour and the reduction of osseous defects on the uprighted teeth were noted [10]. The disadvantages of orthodontic treatment have also been studied. For example, if an uncontrolled inflammation is present around the periodontal tissue, applying orthodontic forces may result in the destruction of surrounding bone [11].

Many studies [7, 12, 13] have been conducted on the advantages of perioorthodontic treatments in certain cases. Brown reported a clinical-histological study on humans and described the effects of uprighting mesially inclined molars with infrabony defects [7]. Corrente et al. performed open flap debridement on teeth with infrabony defects before applying light continuous orthodontic forces [13]. Geraci et al. reported the growth of new connective tissue into a periodontal vertical defect during tooth movement [14].

This case report discusses the treatment of a mesially impacted second molar that shows periodontal destruction using a removable orthodontic appliance in combination with SRP.

## 2. Case Presentation

**Figure 1:**
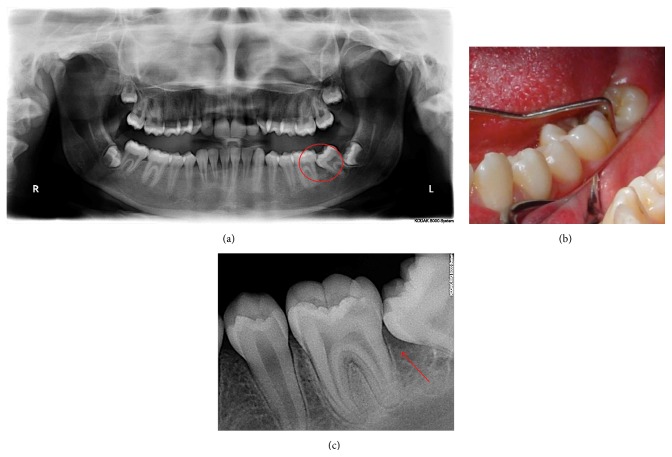
(a) Radiographic view of baseline (red circle showing defect side), (b) clinical view of defect side with periodontal probe, and (c) radiographic view of defect side (red arrow showing bone level).

A 20-year-old systemically healthy female patient was referred to our clinic. The clinical evaluation of the patient revealed low (<20%) full-mouth plaque score and full-mouth bleeding scores [15]. There were no periodontally diseased sites other than the lower-left posterior region of the patient. Clinical and radiological evaluation showed mesial tipping and vertical bone loss caused by impaction of the lower-left second molar. Blood on probing and excessive plaque accumulation were present on distal sites of the first molar and mesial sites of the second molar teeth. The initial probing depth was 8 mm on the mesial surface of the second molar. The distal surface of a neighboring first molar also had a probing depth of 8 mm (Figures 1(a)–1(c)). The malposition of the lower-left second molar was suspected to be the reason of the localized bony defect.

**Figure 2:**
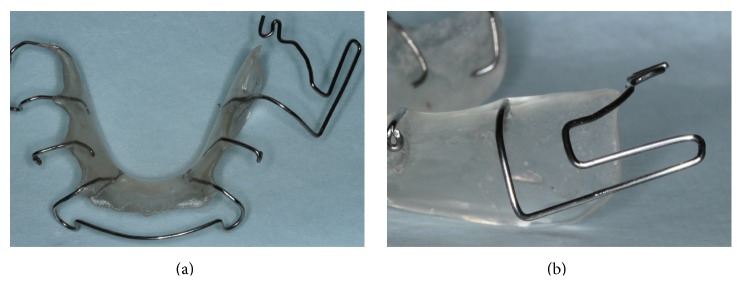
A sample removable appliance explaining T-loop: (a) occlusal view, (b) left view.

**Figure 3:**
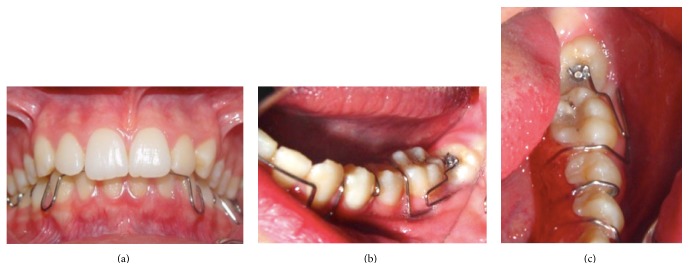
Clinical view after applying appliance: (a) frontal view, (b) left view, and (c) occlusal view.

At first visit, nonsurgical periodontal therapy was performed, and the patient was given oral hygiene instructions to maintain plaque control at the defect site. Three weeks after the initial therapy, a removable appliance with a T-loop was designed to upright the lower-left second molar tooth (Figures 2(a)-2(b)). The uprighting force was transferred to the teeth by a bonded button on the occlusal surface of the lower second molar tooth (Figures 3(a)–3(c)).

The patient was scheduled for follow-up every two weeks to maintain the patient's oral hygiene and the continuous orthodontic force by activating the uprighting spring. Because of the compromised periodontal status of the second molar, approximately 150 grams was applied to the button by activating the spring about 1 mm distally in each visit. The open end of the loop in the removable appliance caused the distal tipping of the tooth as expected. The appliance was used for three months. As the patient's growth and development period had not been completed, the extraction of the third molar was delayed because of the possibility of an acceptable eruption. After the finalization of orthodontic treatment, reevaluation of the third molar showed that a successful eruption was not possible. Thus, the patient's third molar was extracted after orthodontic treatment.

**Figure 4:**
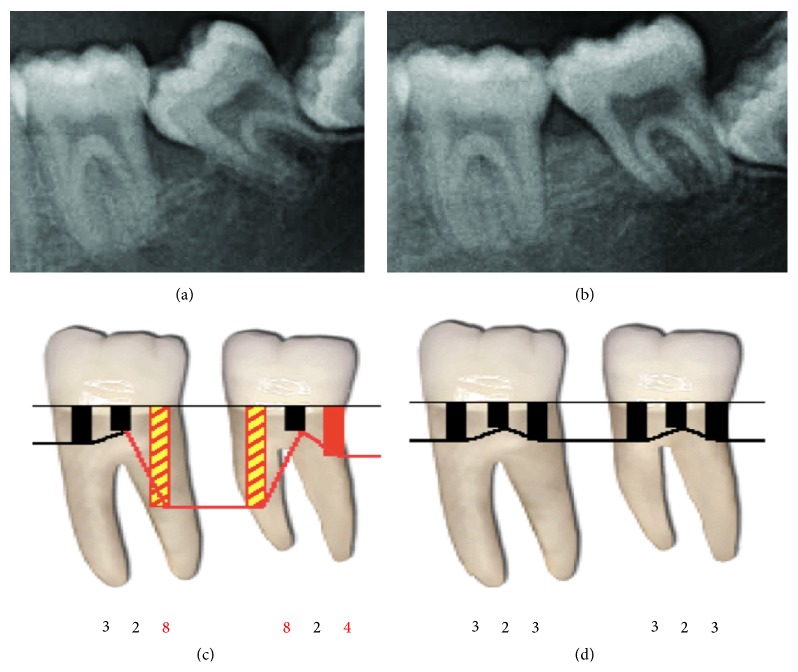
(a) Radiographic view of pretreatment, (b) radiographic view of third month after the treatment, (c) probing pocket depth values of pretreatment, (d) probing pocket depth values after the treatment.

At the end of the orthodontic treatment, the patient showed a significant improvement in the periodontal tissues (Figures 4(a)-4(b)). Gingival tissues around first and second molar teeth healed successfully, showing no signs of inflammation and bleeding on probing. The tooth was positioned in a physiological location, and the probing depth measurements were reduced from 8 mm to 3 mm. Also the remodeling of the bone was achieved by a distal tipping movement (Figures 4(c)-4(d)).

## 3. Discussion

Many host-dependent etiologies [5] can cause the impaction of second molars and iatrogenic situations, such as incorrect positioning of the molar bands or excessive distalization of the first molars during orthodontic treatment [5, 16]. This case report shows that developmental alterations or inconsistencies in the mandibula and dental arch may induce the impaction of the second molar.

Orthodontic separator appliance, surgical repositioning or luxation, extraction of the second molar to enable the eruption of a third molar, reimplantation of the second molar or transplantation of the third molar to the extraction site, and orthodontic uprighting procedures have been suggested as treatment options for impacted second molars [5]. The proper time to treat impaction is early adolescence [7]. In our case, the subject was 20 years old, and the treatment was delayed because of the patient's unawareness of the malposition. This situation may generate complicated periodontal conditions that are difficult to manage because of the type of bony defect in such delayed cases.

The main etiologic factors for this type of localized periodontal disease are increased retention of plaque accumulation and incompatible enamel position [6]. In this type of case, previous studies have suggested the use of periodontal regenerative approaches before orthodontic treatment [8, 13]. However, such limitations as the remaining uncontrollable plaque retention site and the inability to achieve sufficient stability for bone graft and membrane after surgery have made GTR treatment a poor choice to obtain success in this case. Moreover, the risk of bone resorption after a full-thickness flap operation [17] and the need for expensive materials also make surgical periodontal therapy less preferred by patients. In our treatment, we used orthodontic forces for the uprighting and repositioning of the second molar. The success of treating the malposition also affected the periodontal status by eliminating the infrabony defect without surgical or regenerative treatment. Bone apposition occurs as a result of the bending of the alveolar wall produced by the pull from Sharpey's fibers in orthodontic movements [18]. In our case, bone fill in the defect site was noted. The distal tipping of the tooth, which causes stress in the periodontal ligaments on the mesial surface of the second molar, may be the reason for the enhancement of bone filling in the defect site.

Recently Cardaropoli et al. reported successful bone fill in pathologic tooth migration cases, carried out with orthodontic tooth movement and GTR procedures [20]. Sağlam et al. achieved successful bone fill in 6 months in the maxillary sinus floor with a bodily movement of a premolar tooth prior to implant placement [19]. In their clinical study Amato et al. reported successful hard and soft tissue augmentations before implant surgery, with orthodontic forced extractions in 12 months. Our study demonstrated radiographic improvements in periodontal tissues within 3 months. Even though this is an unreliable result due to relatively short time for periodontal tissue regeneration, an immature bone apposition in the bony defect, mesial to the second molar tooth, may explain the radiographically observed bone fill in that time.

Clinical studies examining the effect of orthodontic treatment on patients with infrabony defects are limited and report different results [21, 22]. A case report on teeth with infrabony pockets and pathologic migration showed that orthodontic movement did not have a negative effect on newly regenerated periodontal tissues [23]. Another study showed that orthodontic movement could increase the destruction severity of the connective tissue attachment on teeth with inflamed, infrabony defects [24]. In our case, inflammation control in the periodontal tissues was achieved before orthodontic therapy through nonsurgical initial therapy. Moreover, during orthodontic treatment, no adverse effects or increased periodontal destruction was observed.

Orthodontic uprighting procedures have been consistently used in mesially impacted molars in previous studies [5, 16, 25–28]. Shapira et al. used fixed uprighting springs hooked into the main archwire for distalization anchorage. Sawicka et al. initially used a partially fixed appliance to maintain anchorage for the uprighting of second molars with long cantilever tip-back springs [26]. Celebi used titanium miniscrews for extrusion in combination with a removable appliance in the distal tipping of a mandibular second molar [12]. In our case, we used a removable appliance with a T-loop design to transfer the uprighting force to a bonded button on the occlusal surface of the second molar for three months. Unlike fixed orthodontic approaches, this removable appliance has the following advantages: it requires less time on the chair side, it makes oral hygiene easy to maintain, no special equipment is required, and it is not an invasive treatment as no other teeth are involved. Disadvantages of the removable appliances are dependency on the patient's cooperation and discontinuous heavy forces transferred to the teeth.

Mandibular third molars play a significant role in second molar impaction and its treatment. An erupting third molar may block the space needed for a second molar and cause impaction [5]. Several studies suggest that, during orthodontic molar uprighting procedures, especially if the root formation of the third molar is complete and impaction is severe, third molars may impede the distal tipping of the second molars [16, 29, 30], resulting in the necessity for extraction. By contrast, as stated in a previous study [31], from a biomechanical perspective, leaving the third molar bud may expedite the second molar rotation. In our case, during orthodontic treatment the third molar bud did not interfere with the distalization movement, therefore the patient's third molar was extracted after orthodontic treatment.

Although many studies have discussed orthodontic outcomes in the uprighting of impacted molars, only a few of them have examined periodontal improvements. Vanarsdall reported remarkable improvements in periodontal conditions after the orthodontic uprighting of inclined molars [32]. Brown noted an average of 3.5 mm reduction in periodontal pocket depth in infrabony defects [7]. Wehrbein and Diedrich investigated the effects of molar uprighting on five parameters (probing depth, attachment level, plaque index, sulcus bleeding, and tooth mobility) and reported a significant decrease in probing depth, bleeding on probing, plaque accumulation, and tooth mobility [33]. In our case, we observed a 5 mm reduction in pocket depth and the resolution of an infrabony defect with proper plaque control by the patient.

There are some shortcomings in our case; no surgical reentry, CBCT scan, and histological sampling were available for supporting the periodontal tissue regeneration. Because of the ethical reasons reentry and histologic sampling were not performed in this case. Also CBCT scan was not available because of the preexisting conditions.

## 4. Conclusion

Despite the limitations, the present study demonstrates that the combined orthodontic and periodontal therapy without using a surgical procedure can provide successful bone fill in the bony defects caused by inclined molars.
